# An Object in View

**Published:** 1884-02

**Authors:** 


### AN OBJECT IN VIEW.

The celebrated Dr. Sydenham had a patient which he had long prescribed for. At last Sydenham acknowleged that his skill was exhausted—that he could not pretend to advise him any further. "But," said he, "there is Dr. Robinson, who lives at Inverness (in the south of Scotland), who is much more skilled in complaints of this kind than I am; you had better consult him. I will provide you with a letter of introduction, and I hope you will return much better.

The patient was a man of fortune, and soon took the road; bu traveling was a very different undertaking then from what it is now and a journey from London to Inverness was not a trifling one He arrived, however, at the place of destination, but no Dr. Robinson was to be found, nor had any one of that name been in town. This, of course, enraged the gentleman very much, and he took the read back to London, raging and vowing vengeance on the doctor. On his arrival he vented all his rage on the latter, and abused him for sending him a journey of so many miles for nothing. When his fury was a little abated, Sydenham said:

"Well, now, after all, is your health any better?"

"Better!" said he; "yes, sir, it is better. I am, sir, as well as I ever was in my life; but no thanks to you for that."

"Well," said Sydenham, "you have reason to thank Dr. Robinson. I wanted to send you on an errand with an object in view. I knew it would do you good; in going you had Dr. Robinson in contemplation, and in returning you were equally busy in thinking of scolding me."

